# Diversity and Agronomic Performance of *Lupinus mutabilis* Germplasm in European and Andean Environments

**DOI:** 10.3389/fpls.2022.903661

**Published:** 2022-06-10

**Authors:** Agata Gulisano, Sofia Alves, Diego Rodriguez, Angel Murillo, Bert-Jan van Dinter, Andres F. Torres, Milton Gordillo-Romero, Maria de Lourdes Torres, João Neves-Martins, Maria-João Paulo, Luisa M. Trindade

**Affiliations:** ^1^Wageningen University and Research Plant Breeding, Wageningen University, Wageningen, Netherlands; ^2^DRAT, Instituto Superior de Agronomia, Universidade de Lisboa, Lisbon, Portugal; ^3^Instituto Nacional de Investigaciones Agropecuarias (INIAP), Estación Experimental Santa Catalina, Quito, Ecuador; ^4^Vandinter Semo, Scheemda, Netherlands; ^5^Plant Biotechnology Laboratory, Universidad San Francisco de Quito, Quito, Ecuador; ^6^Wageningen University and Research Biometris, Wageningen Research, Wageningen, Netherlands

**Keywords:** Andean lupin, phenotypic diversity, germplasm characterization, breeding, grain yield, biomass, vegetative development

## Abstract

The introduction of *Lupinus mutabilis* (Andean lupin) in Europe will provide a new source of protein and oil for plant-based diets and biomass for bio-based products, while contributing to the improvement of marginal soils. This study evaluates for the first time the phenotypic variability of a large panel of *L. mutabilis* accessions both in their native environment and over two cropping conditions in Europe (winter crop in the Mediterranean region and summer crop in North-Central Europe), paving the way for the selection of accessions adapted to specific environments. The panel of 225 accessions included both germplasm pools from the Andean region and breeding lines from Europe. Notably, we reported higher grain yield in Mediterranean winter-cropping conditions (18 g/plant) than in the native region (9 g/plant). Instead, North European summer-cropping conditions appear more suitable for biomass production (up to 2 kg/plant). The phenotypic evaluation of 16 agronomical traits revealed significant variation in the panel. Principal component analyses pointed out flowering time, yield, and architecture-related traits as the main factors explaining variation between accessions. The Peruvian material stands out among the top-yielding accessions in Europe, characterized by early lines with high grain yield (e.g., LIB065, LIB072, and LIB155). Bolivian and Ecuadorian materials appear more valuable for the selection of genotypes for Andean conditions and for biomass production in Europe. We also observed that flowering time in the different environments is influenced by temperature accumulation. Within the panel, it is possible to identify both early and late genotypes, characterized by different thermal thresholds (600°C–700°C and 1,000–1,200°C GDD, respectively). Indications on top-yielding and early/late accessions, heritability of morpho-physiological traits, and their associations with grain yield are reported and remain largely environmental specific, underlining the importance of selecting useful genetic resources for specific environments. Altogether, these results suggest that the studied panel holds the genetic potential for the adaptation of *L. mutabilis* to Europe and provide the basis for initiating a breeding program based on exploiting the variation described herein.

## Introduction

*Lupinus mutabilis*, also known as tarwi, pearl, or Andean lupin, is a high protein legume, native to the Andes and currently cultivated almost exclusively in Ecuador, Peru, and Bolivia (Mercado et al., 2018). This species is characterized by the highest grain quality of all cultivated lupins. Its seeds have a protein and oil content similar to that of soybean, 44% dry weight (dw) and 18% dw, respectively, but are also characterized by the absence of starch and the presence of most essential amino acids, including methionine and cysteine (Gulisano et al., 2019). In the past 20 years, *L. mutabilis* has gained increasing interest in Europe, as it could represent a superior alternative to the current plant-based sources of protein and oil, such as soy or pea. The potential for the successful introduction of *L. mutabilis* to Europe is high as the crop is adapted to low input farming and temperate climatic conditions in the Andean region where it originates. Similarly, *L. mutabilis* could contribute to the development of sustainable and competitive biomass industries by increasing biomass supply from marginal lands.

*Lupinus mutabilis* is the only economically important species of the genus *Lupinus* that originates from the New World (South America). *Lupinus albus*, *L. luteus*, and *L. angustifolius* all originated from the Mediterranean region (Wolko et al., 2011) and are the most cultivated worldwide (Abraham et al., 2019). However, old world lupin species are still characterized by relatively low and unstable yields, producing at their best between 2.5 and 4 t/ha and having a lower content of protein (34–42%) and oil (5–11%) in the seeds than *L. mutabilis* (Abraham et al., 2019; Gulisano et al., 2019). The domestication of lupins in the Old and New Worlds was completely independent but followed similar patterns, involving phenotypic changes toward non-shattering pods, permeable seed coats, and large seeds. Demographic analysis suggests that *L. mutabilis* was domesticated in northern Peru around 2,600 years ago, as it split from the wild progenitor *L. piurensis*. *L. mutabilis* was derived from a small subset of the ancestral population, which then went through a classical domestication selection process and a subsequent rapid population expansion as it became cultivated across the Andes (Atchison et al., 2016). The presence of island-like habits and diverse ecological opportunities in the Andean region has led to exceptional rates of diversification in the whole Andean lupin clade (Hughes and Eastwood, 2006). Similarly, *L. mutabilis* is characterized by a high phenotypic diversity that allowed its adaptation to a wide range of altitudes and microhabitats as it expanded from Colombia to the north of Argentina.

At present, *L. mutabilis* remains an understudied crop, inadequately characterized and underutilized. The expansion of its cultivation has been strongly limited by the presence of toxic alkaloids in its seeds and low yields. The potentially high alkaloid levels do not represent an insurmountable barrier since low alkaloid genotypes are already available (reviewed in Gulisano et al., 2019). The main hindrance to the establishment of *L. mutabilis* as a crop in Europe is primarily the species’ low productivity and long vegetation periods. Previous studies have pointed out the importance of breeding for a better plant architecture and early maturity to address these bottlenecks (Caligari et al., 2000). In fact, suboptimal yields are mainly caused by the indeterminate growth habit of the crop, which favors vegetative growth at the expense of seed production. Indeterminate growth is an undomesticated characteristic of many grain legumes and remains a major challenge for legume breeders. The ability to prolong indefinitely the vegetative phase after the onset of flowering is expressed in lupin species through the production of successive orders of branches throughout the cropping season. Indeterminate growth habit has been overcome in *L. albus*, *L. luteus*, and *L. angustifolius* through the selection of spontaneous or induced mutants with a determinate or semi-determinate growth habit (Wolko et al., 2011; Gorynowicz et al., 2014).

The study of the genetic variation present within germplasm collections can support breeding programs in defining useful genetic resources, adaptation strategies, and adaptive traits. The genetic diversity present in *L. mutabilis*, notably its Andean clade, can provide ample resources for breeding programs aimed at enhancing its commercial potential and successful introduction to new production systems. South American institutions started gathering *L. mutabilis* germplasm in 1947 and currently hold the largest and most diverse collections (including more than 3,000 different genotypes) in the gene banks of Peru, Ecuador, and Bolivia. However, a comprehensive study of *L. mutabilis* germplasm from the Andes and its performance across different environments is still lacking. Accordingly, preliminary evaluations of the performance of *L. mutabilis* in Europe have been carried out using a limited set of lines (resulting from mutations and repeated selection) across very similar environments (Caligari et al., 2000; Galek, 2010; Galek et al., 2017; Guilengue et al., 2020). These studies have highlighted the availability of relevant breeding traits within the small panel of investigated genotypes (Guilengue et al., 2020), thus underscoring the importance and necessity of a large-scale evaluation of *L. mutabilis* germplasm and its performance across different environments in Europe.

In this study, we addressed the aforesaid lack of evaluation of wide germplasm collections in different agroclimatic areas, both at a transnational and a transcontinental level. We evaluated the largest collections of *L. mutabilis* accessions under study, comprising accessions from the Ecuadorian germplasm bank of the Instituto Nacional de Investigaciones Agropecuarias (INIAP) and European breeding programs. This large panel of 226 genetically diverse accessions was evaluated for its agronomic performance both in its native environment (Ecuador) and across the two potential cropping conditions in Europe. Field trials were conducted to evaluate the genotypic panel’s suitability as a winter and summer crop in the Mediterranean and North/Central Europe, respectively. Scoring of morphological, phenological, and yield-related traits provides valuable information for germplasm users and serves as the basis for the classification of accessions adapted to specific environments and suitable for seed and biomass production.

## Materials and Methods

### Plant Material

**FIGURE 1:**
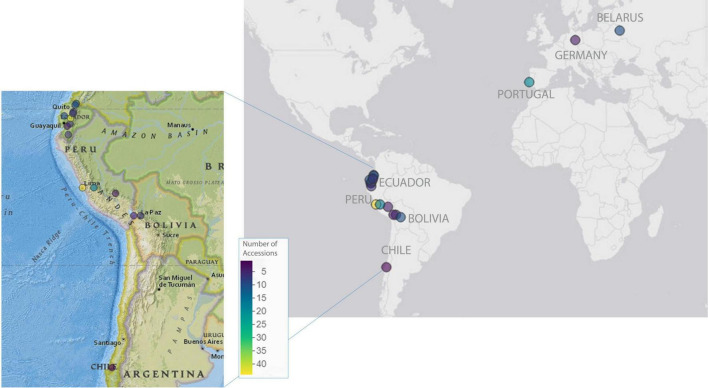
Geographic distribution of the 225 *Lupinus mutabilis* accessions included in the panel under study. Dots on the map indicate the provenience of the accessions. For the breeding material, dots indicate the institutes developing and providing the lines for our collection (ISA, Portugal; JKI, Germany; Byelorussian Agricultural Academy, Belarus). For the accessions from South America (donated by INIAP), dots indicate the provenience of landraces, varieties, and wild material collected along the Andean region, when these data were available. Maps were generated using mapview package in R (v. 2.11.0, Appelhans et al., 2022).

A panel of 225 genetically diverse *L. mutabilis* accessions was used in this study to investigate phenotypic variability within this species and to test *L. mutabilis* in different environments (see Figure 1). A large part of the panel comprised 201 accessions selected from the INIAP germplasm bank and included landraces, varieties, and wild material collected across Ecuador (96), Peru (64), Bolivia (15), and 8 lines donated to the collection by the Byelorussian Agricultural Academy. The origin of 18 accessions remains unknown due to the lack of passport data for these germplasm resources. The panel herein will be further referred to as the GWAS panel. This panel was evaluated in four field trials, including one location in Ecuador and three locations in Europe, representing an example of cultivation in the native environment, winter Mediterranean and summer North/Central European cropping conditions. Two cultivars were included in the study as references: I-450 ANDINO (INIAP-450 ANDINO, 1999) was included in the Ecuadorian field trial, while Inti (Gross et al., 1988) was included in the European field trials. Additionally, 24 *L. mutabilis* lines developed in breeding programs in Europe were evaluated in the European field trials. These lines were provided by the Instituto Superior de Agronomia (ISA, Lisbon, Portugal) and the Julius Kühn-Institut (JKI, Quedlinburg, Germany) and are potentially better adapted to European conditions. A more detailed list of all accessions included in this study is available in Supplementary Table 1.

### Field Characterization

**FIGURE 2:**
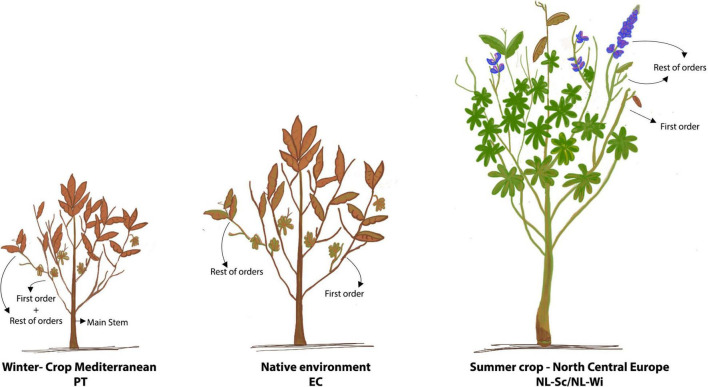
Morphological development of *Lupinus mutabilis* in the tested environments. At harvest, in PT and EC, plants reached full maturity, were dry, and their architecture was generally restricted to two branching orders (semi-determinate growth habit). In NL, at harvest, plants were highly branched, still switching between vegetative and reproductive phases and therefore holding floral buds and pods often no economic value (indeterminate growth habit). In evidence, the different architectural parts of the plant are the main stem (MS), the first branching order (FO), and the rest of the branching orders (RO). In PT, production on the first order and the rest of the orders were scored together.

The effects of genotype, environment, and genotype by environment (G × E) interactions on the phenotypic variation of morphological traits were assessed on four locations, one in Lisbon in 2018 (PT), two in Netherlands, respectively, in Scheemda in 2019 (NL-Sc) and in Winschoten in 2020 (NL-Wi), and one in Cotopaxi, Ecuador in 2020 (EC). In Europe, field trials were grown during winter for the Portuguese site, and during summer in Netherlands, on clay soil in 2019 and on sandy soil in 2020. In Ecuador, *L. mutabilis* accessions were sown in December 2019 and harvested in June 2020, following local cultivation practices. In all locations, plants were cultivated under rain-fed conditions and without the aid of any fertilization (refer to Table 1). In Europe, a randomized complete block design in three replicates was used. The experimental units were plots, including 20 plants (4 rows with 5 plants per row), at a distance of 30 cm × 30 cm. Phenotyping of morphological and phenological parameters was conducted only on the six central plants of the plots (biological replicates). In Ecuador, an Alpha Lattice design (40 × 5) in three replicates was used. The experimental units were rows of 2 m, with 10 sowing spots at a distance of 20 cm, and a distance of 80 cm between plots. Phenotype was scored on the 5 central plants. Quantitative traits related to plant morphology, phenology, and agronomic performance were measured in all the locations. The traits to phenotype were adapted from the IBPGR descriptors [IBPGR, 1981] based on the feasibility of their scoring on a large scale and keeping in mind the main breeding goals for *L. mutabilis*, namely earliness, plant architecture, grain, and biomass yield. Morphological measurements included plant height (scored at harvest), fresh biomass (fresh weight of the total biomass harvested, after eliminating the root system), number of branching orders (0 = main stem only, 1 = main stem and first branching order, etc.), and number of pods and seeds present, respectively, on the main stem (MS), on the first branching order (FO), and on the remaining branching orders (RO), as illustrated in Figure 2. In Portugal, due to the restricted architecture of the plants, the scoring of pods and seeds was divided only over the main stem production and the remaining part of the plant (mainly first-order branches). Phenology of the accessions was investigated by scoring germination and flowering time (as number of days from sowing) and calculating growing degree days (GDD) accumulation using 4°C as the baseline temperature (as in Hardy et al., 1998). Due to the impossibility of collecting data during COVID-19 restrictions, measurements of phenological traits in Ecuador were not possible. Finally, agronomic performance assessment included measurements of the total number of pods and seeds harvested, 100 seeds weight (dry), and aboveground biomass fresh weight (g). Seed yield was measured per plant at harvest after drying the seeds at room temperature (dw, g/plant), while vegetative yield (dw, g/plant) was estimated as the difference between the total amount of biomass harvested (dw) and the seed yield per plant.

**TABLE 1:** Environmental characteristics of the four field trial locations during the respective growing season.

		EUROPEAN ENVIRONMENTS		
Trial name	EC	PT	NL-Sc	NL-Wi
Location	Cotopaxi, Ecuador	Lisbon, Portugal	Scheemda, Netherlands	Winschoten, Netherlands
Coordinates	0°55′35.1″S 78°40′07.4″W	38°42′33.5″N, 9°11′00.5″W	53°09′60.00″ N, 6°57′59.99″ E	53° 10′ 11.346, 7° 2′ 56.096″
Altitude (m)	2,948	60	−1	3
AverAGE Temperature (°C)	13.6	15	14	15
TOTAL Precipitation (*mm*)	854.7	260.1	328.7	408.8
Day length (hours)*	12	10→ 14.2	14 → 10.44	14 → 10.44
MAximum daY Length (hours)	12	14.2	16.5	16.5
AVERAGE Relative Humidity (%)	86%	74%	77%	75%
AVERAGE Wind speed (m/s)	5.2	21.7	4.24	15
Growing Season	December 2019–June 2020	November 2018–May 2019	April–October 2019	April–October 2020
Soil Type	Sandy	Clay	Clay	Sandy

*Photoperiodic differences in the different environments are reported, indicating the change in the amount of daylight hours from sowing to harvest time.

### Statistical Analysis

Single-site analyses were conducted in each environment, using the R package *statgenSTA* (van Rossum et al., 2021). A linear mixed model with spatial correction was fitted for each trial separately and used to estimate adjusted means. The model fitted with the R package *statgenSTA* (version 1.0.8) included a fixed effect for genotype, a fixed effect per block, and an extra spatial component obtained using SpATs, which uses two-dimensional smoothing with P-splines as described in Rodríguez-Álvarez et al. (2018). By fitting a smooth surface through joint modeling of additive one-dimensional trends plus interactions between trends in the row and column directions, this method can account for all sources of continual environmental variation and explicitly model both large-scale and small-scale spatial dependences, leading to an improvement in precision and predictions of genotypic values (Velazco et al., 2017; Rodríguez-Álvarez et al., 2018). Adjusted means, Best Linear Unbiased Estimators (BLUEs), were obtained from the same model for each line in each environment. BLUEs were used to perform the Principal Component analysis per site using the *FactoMineR* package and to calculate phenotypic correlation between traits using the *cor* function of base R. For the estimation of heritability, the same mixed model fitted with *statgenSTA* was used but treating genotypes as random factors.

## Results

### Cropping Conditions Highly Impact Morphology and Yield *of Lupinus mutabilis*

A large and diverse panel of *L. mutabilis* accessions was used to test the agronomic performance of this species under three different cropping conditions, two in Europe and one in the Andean region (Ecuador), the native environment of this species. In Europe, it has been tested as a winter crop in the Mediterranean area (Portugal) and as a summer crop in North-Center Europe (Netherlands) both on sandy and clay soils. The overall performance of *L. mutabilis* showed extreme variation between the different environments tested in Europe and between European and Ecuadorian environments, in terms of yield, vegetative development, and phenology (refer to Figure 2 and Table 2). In terms of grain yield, the best performance was observed in Portugal (on average 18.1 g/plant), followed by Ecuador (9.2 g/plant), and the Dutch site on sandy soil with 6.6 g/plant. The lowest yield was measured in the field trial in Netherlands with clay soil (seed yield of 2.5 g/plant on average). This agronomic variation can be explained by the different climatic and soil conditions characterizing each environment. When growing on clay soil in winter Mediterranean conditions (Portugal), *L. mutabilis* stayed generally smaller than in Ecuador, reaching a height of 60 cm and producing the lowest amount of biomass (116 g/plant). Low precipitation and high temperatures after flowering contributed to seed maturation in this environment and restricted biomass accumulation due to drought. It is also worth noting that in Portugal, plants were slightly more branched than in Ecuador and developed more often a second branching order (1.6). On the contrary, summer cropping conditions in Netherlands prompted a higher vegetative development and indeterminacy, which severely impacted grain yield. With higher precipitation, *L. mutabilis* accessions grew taller (∼90 cm) and developed more than two branching orders (1.88–2.65), generating an increase in the production of above-ground biomass. At the Dutch site with clay soil (NL-Sc), the performance was generally poorer, and plants were characterized by both the lowest grain and biomass yield. Short days in Portugal did not significantly affect flowering time (110 days), which was in line with the general flowering time observed in Ecuador under a fixed photoperiod. Conversely, long days in Netherlands remarkably shortened flowering time by about a month (to 80–90 days). However, in both European environments, it was possible to distinguish earlier and late flowering accessions characterized by a different growing degree day (GDD) accumulation. In Portugal, early accessions had a GDD of about 600°C and flowered before 105 days after sowing, while late accessions flowered between 116 and 127 days with a GDD of about 1,000°C. In Netherlands, some accessions were very early and flowered between 73 and 80 days after sowing (GDD ≈700°C), while a higher proportion was late and flowered between 90 and 104 days (GDD ≈1,200°C).

**TABLE 2:** Mean, range of variation, and broad-sense heritability (H^2^) for the BLUEs values of morphological and phenological traits were assessed across the different field trials.

	Native environment			Winter crop- Mediterranean			Summer crop- North-Central Europe					
	EC			PT			NL-Sc			NL-Wi		
	Mean	Range	H^2^	Mean	Range	H^2^	Mean	Range	H^2^	Mean	Range	H^2^
Germination time (days)	−	−	−	10.8	(8.06, 16.7)	**0.51**	−	−	−	17.8	(11.6, 32.0)	**0.78**
Height MS (cm)	73.6	(24.2, 95.9)	**0.72**	59.9	(27.5, 104)	**0.86**	90.6	(41.3, 208)	**0.56**	92.7	(47.5, 121)	**0.57**
Flowering time (days)	−	−	−	110	(91.0, 127)	**0.93**	90.8	(75.6, 113)	0.24	82	(65.8, 98.6)	**0.87**
Fresh biomass (g)	−	−	−	116	(4.45, 240)	**0.68**	507	(132, 1,910)	0.19	750	(93.7, 2,010)	0.32
Branching Orders	1.09	(0.225, 1.97)	0.33	1.6	(0.798, 2.18)	**0.44**	1.88	(0.868, 2.78)	**0.46**	2.65	(1.34, 3.93)	0.07
Pods MS	8.74	(4.76, 17.5)	**0.48**	12.8	(5.20, 26.2)	**0.42**	2.84	(0.023, 13.4)	**0.83**	2.84	(0.023, 13.4)	**0.46**
Pods FO	7.17	(0, 27.2)	0.18				7.79	(0, 20.7)	**0.54**	11.9	(0, 44.3)	0.39
Pods RO	0.29	(0, 7.07)	0.07	24.1	(8.27, 48.9)	**0.43**	3.38	(0, 18.3)	0.39	5.51	(0, 47.3)	0.12
Pods T	16.1	(2.34, 41.3)	0.23	36.1	(19.7, 70.7)	**0.41**	7.79	(0, 20.7)	**0.69**	19.7	(5.29, 62.1)	**0.58**
Seeds MS	25.2	(10.9, 56.0)	**0.46**	42.3	(17.0, 67.4)	**0.57**	13.9	(0.287, 39.7)	**0.83**	19.9	(5.02, 51.8)	**0.53**
Seeds FO	16.2	(0, 73.7)	0.15				14.7	(0, 55.3)	**0.55**	18.3	(0,120)	**0.62**
Seeds RO	0.374	(0, 14.9)	0.07	54.7	(22.3, 108)	0.33	6.32	(0, 26.5)	0.38	0.856	(0, 20.8)	−
Seeds T	41.6	(3.74, 119)	0.21	95.4	(50.9, 158)	0.36	26.4	(0.839, 71.7)	**0.70**	41.7	(5.30, 175)	**0.64**
100 Seed weight (g)	23.2	(9.70, 30.1)	**0.67**	25.3	(7.63, 59.9)	0.17	18.6	(8.13, 28.8)	**0.63**	42.1	(11.2, 145)	0.24
Seed yield (g/plant)	9.16	(0.3, 25.9)	0.15	18.1	(6.5, 32.5)	0.28	2.5	(0.5, 11.3)	0.32	6.57	(0.5, 16.5)	0.37
Vegetative yield (g/plant)	−	−	−	45.8	(15, 97.8)	0.54	133.6	(30.7, 643.11)	0.17	174.9	(13.5, 451.2)	0.21

*Values in bold signify a relatively high heritability.*

### High Variability in the Studied Panel for Several Morphological Traits

Phenotypic evaluation of the *L. mutabilis* panel revealed significant variation in many of the measured traits, confirming the presence of significant variability within the studied panel of accessions. Raw data were first corrected for spatial variation within each trial, obtaining BLUE values for each trial that were used in further analysis. Spatial variation is common in field trials, and accounting for it increases the accuracy of estimated genetic effects (Selle et al., 2019). After the correction, the variability observed was decomposed into genetic, spatial, and residual variance, and heritability values were estimated. Table 2 summarizes the BLUEs and heritability values estimated for each trait in each of the tested environments. A large range of variation for the scored traits pointed out a high variability between accessions, especially for yield-related traits, such as production of pods and seeds on the first branching order and on the rest of the plant (refer to Table 2). High heritability for plant height (0.5–0.9) and seeds and pods production on the main stem (0.4–0.8) were observed in all the field trials. Flowering time, which is a genetically determined trait, was highly heritable (0.9) both in PT and NL-Wi. The low value of heritability reported for flowering in NL-Sc is explained by the high amount of variance associated with residual and spatial variation (refer to Supplementary Table 2), probably caused by the large influence that climatic conditions had on flowering in this location. Conversely, the estimation of higher heritability for total number of seeds and vegetative yield in the environments where the mean estimates for these traits were lower (Portugal and Netherlands, respectively, refer to Table 2) can be explained by the smaller range of phenotypic variation assessed and associated with spatial variation (refer to Supplementary Table 2). Limiting environmental conditions for these traits, such as drought for vegetative yield in PT and abundant precipitations for seed yield in NL, contributed to even out phenotypic variation within accessions and resulted in a higher variation between accessions, resulting in higher heritability estimates (refer to Supplementary Table 2). Relatedness between accessions and contributions of single traits to the total variance were estimated *via* a principal component analysis for each trial. Flowering time, yield-related traits (seed yield, vegetative yield), and architecture-related traits (number of branching orders and number of seeds from the different branching orders) were the main factors contributing to principal components and explaining the larger fraction of variation between *L. mutabilis* accessions performance in each environment.

### Variability in Ecuador Is Largely Due to Yield and Plant Architecture-Related Traits

**FIGURE 3:**
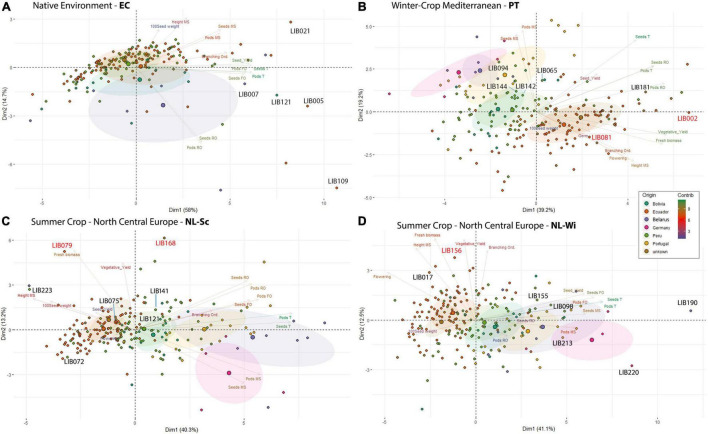
Principal component analysis of *L. mutabilis* collection, including 201 lines from the INIAP gene bank (Bolivia, Ecuador, Peru, unknown, and Belarus), Andino, and 24 lines from breeding programs in Europe (Germany and Portugal). Each biplot shows the PCA scores of the explanatory variables (as vectors) and individuals (as points) separately for each of the environments tested: **(A)** Ecuador, **(B)** Portugal, **(C)** NL-Sc and **(D)** NL-Wi. Individuals on the same side as a given variable should be interpreted as having a high contribution to it. The color of the explanatory variables (vectors) shows the strength of their contribution to each PC. The five most high-yielding genotypes for each location are indicated on the graph with a black label. The accessions with the higher biomass yield in European trials are indicated in red (reported in Table 4).

In Ecuador (Figure 3A), the variability among accessions was explained mainly by the total number of pods/seed and the share of grain production on the first branching order in PC_1_ (58%) and by the share of production on the remaining branching orders in PC_2_ (14.7%). In their native environment, the majority of *L. mutabilis* accessions were characterized by low scores for total seed yield, primarily from the first branching orders (clustered on the left side of the graph, Figure 3A). In opposition, clustered on the right side of the graph, we found the accessions with higher grain yield and a few accessions characterized by a particularly high production on the secondary branching orders (bottom right) and on the main stem (top right). The five accessions with higher grain yield produced between 22 and 26 g/plant in this environment (as listed in Table 3) and included both lines characterized by a higher yield on the main stem (LIB021) and first branching orders (LIB007) and lines characterized by a high grain production coming from higher branching orders.

**TABLE 3:** BLUEs data for seed yield (g/plant) and flowering time (days from sowing) of the five most yielding accessions in each environment.

	Ecuador				Portugal					Netherlands- Sc			Netherlands- Wi			
	Geno	Origin	Seed yield	Flowering time	Geno	Origin	Seed yield	Flowering time	Geno	Origin	Seed yield	Flowering time	Geno	Origin	Seed yield	Flowering time
1	LIB109	Ecuador	25.9	−	LIB065	Peru	32.4	102	LIB072	Peru	11.26	88	LIB155	Peru	16.5	68
2	LIB021	Ecuador	23.9	−	LIB142	Peru	26.7	107	LIB223	Peru	7.5	95	LIB098	Peru	15.3	79
3	LIB005	Ecuador	22.8	−	LIB144	Peru	26.6	105	LIB075	Ecuador	6.2	108	LIB220	Germany	14.2	71
4	LIB007	Ecuador	22.4	−	LIB181	unknown	26.3	115	LIB121	Bolivia	5.8	99	LIB213	Portugal	14.2	68
5	LIB121	Bolivia	22.1	−	LIB094	Peru	26.1	97	LIB147	Peru	5.7	84	LIB190	Belarus	13.5	69

### Two Different Morphotypes Are Identified Under European Cropping Conditions

In European environments (Figures 3B–D), it is possible to observe a more defined clustering around germplasm pools of accessions of the same origin that exhibit similar phenotypes. In Mediterranean winter cropping conditions (Figure 3B), almost 80% of the variation observed across the panel was explained by the first three principal components. PC_1_ explained 39.2% of the variation by opposing accessions with larger coefficients for traits, such as fresh weight and seed yield, from secondary branching orders on the right, to smaller accessions with a seed yield mainly concentrated on the main stem on the left. PC_2_ explained 19.2% of the variance, dividing on the vertical plane landraces collected across South America characterized by later and taller plants, from accessions resulting from previous breeding programs in Portugal, Belarus, and Germany characterized by a higher production of seeds on the main stem. On the contrary, in both trials in Netherlands (Figures 3C,D), five dimensions were retained to explain 80% of the variance. Around 40% of the variation was already explained in the first dimension that opposed accessions based on their seed production concentrated on the main stem and first branching order. Instead, the second dimension divided accessions mainly on the basis of their vegetative development, opposing accessions characterized by high biomass yield, and indeterminate growth (top) to smaller and more determinate accessions (bottom). Similarly to Portugal, we observed a higher vegetative development and lower seed yield for accessions from the INIAP collection and a higher seed yield accompanied by a restricted development for breeding lines developed for European environments. However, in this environment, accessions from Peru showed a certain proximity to breeding lines, leading in certain cases to even superior yields. This distinction was particularly evident in the trial on sandy soil (NL-Wi, Figure 3D), where the presence of favorable conditions increased the discrepancy in performance between lines from European breeding programs and other germplasm material (more sparse points). Notably, the five top grain yielding accessions (listed in Table 3) differ in each environment. Similarly to Ecuador, in Portugal, top-yielding genotypes include both lines characterized by a higher yield on the main stem and lines characterized by high grain production coming from higher branching orders. However, it is important to consider that in these environments the seed production scored as coming from the other orders (thus excluding the main stem production) is in fact coming only from the first and second branching orders due to the limited branching observed in these conditions. Conversely, in Netherlands, where seed yield was distributed among more branching orders, the highest yielding accessions had higher seed production on the main stem and on the first branching order. For the European trials, Table 4 lists the two top-yielding accessions in terms of fresh biomass and vegetative yield for each environment, and biomass data are also reported for the top grain-yielding accessions presented in Table 3.

**TABLE 4:** BLUEs data for fresh biomass yield (fresh weight of total aboveground biomass, g/plant) and vegetative yield (dry weight of stems and leaves, g/plant) in Europe.

	Portugal				Netherlands- Sc				Netherlands- Wi			
	Geno	Origin	Fresh Biomass	Vegetative yield	Geno	Origin	Fresh Biomass	Vegetative yield	Geno	Origin	Fresh Biomass	Vegetative yield
1	LIB081	Ecuador	239.9	74.2	LIB079	Ecuador	1,907.3	489.6	LIB017	Ecuador	2,012.6	367.3
2	LIB002	Ecuador	227.6	89.4	LIB168	unknown	1,430.8	424.2	LIB156	Ecuador	1,759.1	423.4
(1)	LIB065	Peru	114.5	50.3	LIB217	Portugal	356.4	173.8	LIB155	Peru	756.7	170.2
(2)	LIB142	Peru	111.7	53.7	LIB216	Portugal	417.6	118	LIB098	Peru	543.3	163.5
(3)	LIB144	Peru	110.8	33.7	LIB220	Germany	206.4	102.3	LIB220	Germany	684.1	126.6
(4)	LIB181	unknown	188.1	62.6	LIB049	Peru	415.7	118.8	LIB213	Portugal	521.2	100.2
(5)	LIB094	Peru	86.6	41.2	LIB130	Peru	561.2	121.4	LIB190	Belarus	328.3	121.5

*In order, the two higher yielding accessions in terms of biomass in each environment, followed by the five accessions with higher grain yield.*

### Prolonged Vegetative Development Negatively Affects Grain Production

**FIGURE 4:**
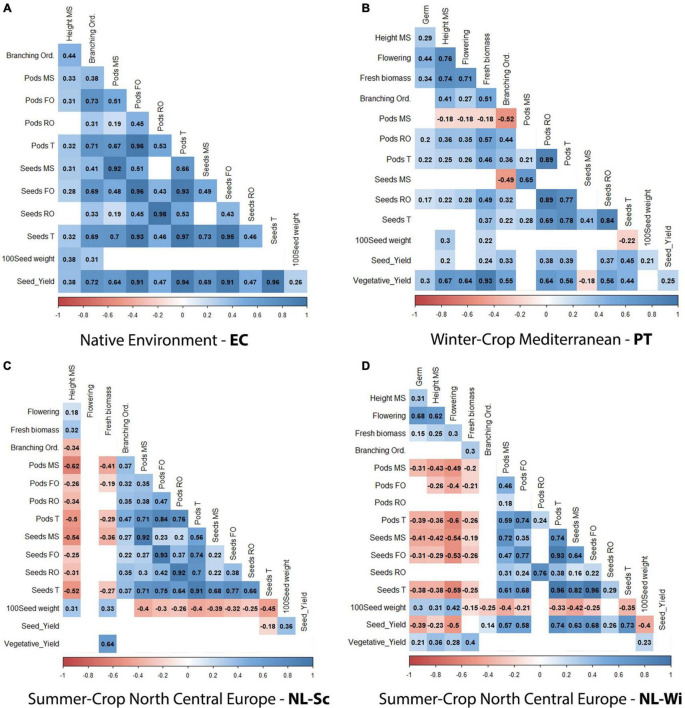
Correlation analysis between scored phenotypic traits (BLUEs data), presented per single field trial: **(A)** Ecuador, **(B)** Portugal, **(C)** NL-Sc, and **(D)** NL-Wi. Red and blue squares indicate significant positive or negative correlation (*p*-value < 0.01), whereas blank cells indicate no significant correlation (*p*-value > 0.01).

BLUEs data were used to investigate correlations between traits (refer to Figure 4). Significant correlations were found across trials, indicating that the total number of seeds produced was mainly correlated with the production on the first branching order (*r*^2^ = 0.77–0.96), followed by the production on the main stem (*r*^2^ = 0.41–0.82) and only to a lower extent with the production on the rest of the branching orders (*r*^2^ = 0.3–0.66). Only in PT, where the count of seeds on the rest of the orders included seeds produced on the first branching order, the correlation with the total number of seeds was higher (*r*^2^ = 0.84, Figure 4B). Conversely, grain production is negatively correlated to vegetative development (height, fresh biomass) in North-Central European conditions, while in PT, only grain production on the main stem appears to be negatively correlated to an increasing number of branching orders. Furthermore, flowering time was highly correlated with vegetative development in PT, where later-maturing accessions produced a higher amount of biomass yield (*r*^2^ = 0.64–0.71, Figure 4B) and negatively correlated with seed yield in NL-Wi, where later-maturing accessions had a lower seed yield (*r*^2^ = –0.5, Figure 4D). In Ecuador, all correlations were positive.

## Discussion and Conclusion

The importance of exploring genetic diversity for the development and selection of superior genotypes is the basis of many breeding programs. To our knowledge, this is the first study exploring the genetic variation and transcontinental performance of an extensive panel of 225 *L. mutabilis* accessions on four geographically distinct trials, including the native Andean environment and two potentially favorable cropping conditions in Europe. The panel of genotypes selected includes for the first time both a wide share of the germplasm collected in the Andean region (201 accessions) and a collection of lines derived from breeding programs taking place in Europe (24 lines). Till present, the lack of such in-depth, wide-ranging studies has represented a limiting factor for the optimization of *L. mutabilis*, hindering its adoption on a commercial scale. Previous work has focused on assessing variation within small pools of genotypes, mainly generated by induced mutations or as a product of crossing (Caligari et al., 2000; Clements et al., 2008; Galek et al., 2016, 2017; Guilengue et al., 2020). As a result, the investigation of the performance and genetic variation available in wider germplasm collections has been highly recommended as a key approach in the generation of tools for crop improvement (González-Andrés et al., 2007; Atnaf et al., 2015; Mousavi-Derazmahalleh et al., 2018). Moreover, the study of germplasm collections across different environments can play an important role in identifying useful genetic resources for targeted breeding and in achieving adaptation to local climatologic conditions (Annicchiarico et al., 2010).

Taking as a reference the behavior of *L. mutabilis* Andean germplasm in its native environment, we observed a very high variation in the agronomic performance of this species in contrasting environments in Europe. Furthermore, germplasm pools summarize a reasonably high portion of the observed variation and are therefore of practical interest for identifying genetic resources, which are likely to possess high adaptation to local conditions or other desired breeding traits. Interestingly, we observed similar range of diversity for Andean germplasm in the native environment, while in European conditions, a clear separation between the performance of Ecuadorian and Peruvian material is noted. These findings reinforce the importance of evaluating large germplasm collections in contrasting environments representing major growing conditions and underline the importance of selecting useful genetic resources for a breeding program on the basis of landrace evaluation in the targeted environment, as also discussed by other authors (Annicchiarico et al., 2010).

In winter Mediterranean cropping conditions, higher temperatures and water scarcity highly affected the architecture of all the accessions, limiting crop development to two branching orders and increasing yield overall in the panel (> 18 g/plant). These observations are in line with previous studies reporting a high effect of temperature and water availability on the degree of determinacy of *L. mutabilis* in Mediterranean conditions, as plants tend to accelerate maturation under dry conditions (Hardy et al., 1997). Even though the environment has a main effect on the architecture of the crop, genotype per environment interactions remain relevant and point out the better adaptation of certain accessions to Mediterranean conditions. Landraces from Peru appear as promising germplasm material to increase grain yield in Mediterranean cropping conditions, as they outperform ISA lines selected specifically for this environment. We reported four Peruvian landraces (LIB065, LIB142, LIB144, and LIB094) producing more than 26 g/plant of dry seeds, which could lead to the grain yield close to or above 3 t/ha, very similar to the values of *L. albus* grain yields growing under the same cropping conditions (López-Bellido et al., 2000). These selected accessions are all characterized by very early flowering (97–106 days), short stature (60 cm), and generally by the development of a single branching order. The highest yielding accession, LIB065, reached a yield of 32.4 g/plant by having both a high seed production and a higher seed weight (30 g/100 seeds). The high performance of this accession can be explained by its origin in the Mantaro valley (Peru), an area characterized by a dry climate where crops are adapted to cultivation under rain fed conditions with drought as the main limiting factor (Silva et al., 2010). The precise origin of the other Peruvian accessions is not known, but they have likely been collected from the highlands of Peru, where ecotypes have been described as small, scarcely branched, and early flowering (Chalampuente-Flores et al., 2021). Instead, ISA breeding lines exhibited a slightly lower branching degree than the Peruvian lines, but their yields were lower, ranging between 7 g/plant (LIB202) and 25.3 g/plant (LIB204). Following LIB204, LIB214 (23.3 g/plant), and LIB212 (21.5 g/plant) appear to be the best ISA accessions for this environment, confirming their good agronomic performance in previous studies (Guilengue et al., 2020; Lazaridi et al., 2020). Therefore, our results expand upon prior work, pointing out the possibility of improving the performance of already selected lines for Europe by making use of the high breeding potential of germplasm from Peru. Notably, a later genotype of unknown origin and characterized by a higher degree of branching orders (LIB181) was also clustered within the genotypes with the highest yield. This supports the prior hypothesis (Martins et al., 2016) that in unstable climates, such as the Mediterranean one, plants with an indeterminate growth might have the advantage of allowing a better compensation of yield when adverse climatic conditions affect pod set, therefore guaranteeing a higher yield.

In North European summer cropping conditions, more constant and higher precipitation prompted *L. mutabilis* vegetative growth throughout the entire cropping season, resulting in highly branched plants holding floral buds and pods of no economic value at harvest. As observed in other legumes, an indeterminate growth habit highly affects the productivity, making the difference between potential productivity and actual yield striking (Sinjushin, 2015). Hence, it is not surprising that accessions with a semi-determinate growth habit (specifically, European breeding lines and Peruvian landraces) were performed best in the Northern European environments. In fact, the determinacy characteristic of these accessions enforces a switch toward the productive phase, which guarantees a more stable seed yield in this environment. Notably, previous evaluations of ISA line LIB213 have reported very late flowering and low productivity in Mediterranean environments (Guilengue et al., 2020). Instead, in northern European environments, we reported relatively high yield and early flowering for this accession. A similar pattern was also observed for lines LIB220 and LIB223. LIB220, which has been selected as a semi-determinate growth type and has been characterized as non-suitable for cultivation in the Mediterranean area (Lazaridi et al., 2020), showed a better adaptation in the north of Europe, in NL-Wi. LIB223, described in literature as a long life-cycle indeterminate accession in Mediterranean conditions (Lazaridi et al., 2020), was actually within the most yielding genotypes when encountering unfavorable conditions in North European conditions (NL-Sc). Overall, the difference in grain yield across the two trials in Netherlands was striking, and very large differences in seed yield were observed for many lines, including top-yielding accessions. On average, the yield on sandy soil exceeded the yield on clay soil by 65%. Lupins characteristically grow on well-drained acidic to neutral soils and are generally intolerant of waterlogging. Waterlogging is common in clay soils and has been found to affect root growth and plant development (Dracup et al., 1998). However, the striking difference in grain yield can also be explained by the amount and distribution of rainfall. Studies on the effect of rainfall on the development and yield of blue lupin in temperate climates have shown that seed yield is dependent to the largest extent on the amount of rainfall in June and July, which are the periods of blooming and pod setting (Podlesny and Podlesna, 2011). The scarcity of rainfall registered for this period in 2019 (2.25 times less than in 2020) might have therefore been the main reason behind the significant decrease in yield observed in NL-Sc (see Supplementary Figure 1).

Considering these results, the use of Peruvian germplasm as the basis for breeding programs aimed at increasing seed yield in *L. mutabilis* for European cropping conditions is recommended. However, the use of Ecuadorian and Bolivian germplasm remains highly valuable for the generation of variation in breeding germplasm and for the selection of new genotypes adapted to Andean conditions. In Table 3, we presented a selection of landraces collected between 2,600 and 3,000 m of altitude, which are best adapted to the conditions of Cotopaxi (at 2,948 m) and produce more than 20 g of seed/plant. These grain yields are superior to the ones of selected varieties, such as I-450 ANDINO (13 g/plant) or LIB091 [17 g/plant; reported as ECU-2658 in Guaytarrilla and Falconí (2014)], and offer the possibility to further increase grain yield *via* breeding. Notwithstanding, our indications on useful genetic resources cannot be considered as conclusive because the lack of repetition in time of the trials prevents assessing the bias derived by genotype × year interactions. However, various features of the germplasm pools, such as higher determinacy of Peruvian and European breeding lines, higher biomass production of Ecuadorian material, and earliness of Peruvian and Bolivian landraces, support previous findings (Gross and von Baer, 1981; Schoeneberger et al., 1982). Hence, these factors have implications for establishing breeding programs for specific locations and increasing the efficiency of genetic resource evaluation aimed at the identification of elite parental material.

Our results clearly indicate that grain yield and architectural traits are strictly correlated, as the highest yielding accessions had a semi-determined growth type, with the main part of the production coming from the main stem (20–50%) and the first branching order (50–80%). Our findings corroborate previous studies that observe high correlations between seed yield and production of pods and seeds in the main stem and first branching orders, concluding that the selection of semi-indeterminate accessions with restricted branching is pivotal to achieving high yield stability in this species (Caligari et al., 2000; Clements et al., 2008; Guilengue et al., 2020). Recessive genes responsible for the restriction of branching have been identified in several lupin species, and the inheritance of this character has been confirmed as monogenic recessive also for *L. mutabilis* (Römer, 1994). Notably, stem height and production of seeds and pods on the main stem appeared as highly heritable across trials, making them good targets for breeding. Although the use of two different experimental designs in Ecuador and Europe, estimations of highly heritable traits were fairly consistent across trials. An accurate estimation of genotype and environmental effects, generally considered more precise on large trials when using incomplete designs (Kumar et al., 2020), was here guaranteed for the plots with a RCB design through the use of spatial correction. In agreement with other studies, we observed high heritability values for flowering time (Harzic et al., 1996). Flowering time was very important for adaptation to both European cropping conditions and appeared negatively correlated to seed yield (*r*^2^ = –0.5). Similarly to what was observed in *L. angustifolius* (Reader et al., 1995) and *L. albus* (Christiansen and Jørnsgård, 2002), time to flowering was reduced when plants were exposed to increase in day length (Netherlands, 70–90 days). Instead, in Portugal under short days, flowering time remained similar to that observed in the Andean region (90–120 days). Flowering time in other lupin species has been shown to be highly responsive to both day length and temperature (Reader et al., 1995; Adhikari et al., 2012). Previous reports on the photoperiodic sensitivity of *L. mutabilis* are contrasting (Hackbarth, 1961; Jacobsen and Mujica, 2008), but flowering time has been reported as highly responsive to the environment, with a predominant effect of temperature (Hardy et al., 1998). Our results suggest that thermal accumulation might play an important role in flower initiation and classify early accessions with a GDD sum of 600°C–700°C and late genotypes with a GDD of about 1,000°C–1,200°C. Similar flowering range have been reported on a small subset of this panel propagated in Portugal in 1994–1995 (Hardy et al., 1998). Even though further studies are needed to clarify the mechanisms regulating flowering time in *L. mutabilis*, our findings indicate that a genotype-specific number of heat units above the base temperature is required to start flowering. Hence, these findings can be useful in the selection of accessions for specific environments. Early accessions are preferable in winter Mediterranean conditions owing to the shorter growing season and to the possibility of escaping terminal drought and heat stress, reducing loss in yield. In North Europe, earlier accessions performed better under optimal conditions, but intermediate flowering resulted in a higher yield when the conditions were suboptimal.

Our observations show that this *L. mutabilis* panel holds the genetic potential for the adaptation of this crop to European environments. The comparison between *L. mutabilis* agronomic performance in its center of origin and two different environments in Europe highlights potential grain yields of up to 3 t/ha in Mediterranean winter conditions, superior to the one achieved in the native region. The capacity of producing such a grain yield in poor soils and under rain-fed conditions, confirm *L. mutabilis* as a potent candidate for sustainable protein production in Europe, and a crop with potential for marginal land. Conversely, the agronomic performance observed in North European conditions confirms the findings of Caligari (Caligari et al., 2000), who singled out indeterminate growth habit as the main factor limiting the yield of *L. mutabilis* in North-European field trials. This suggests that semi-determinate types remain preferred to ensure stable yields, while grain yield, earliness, and plant architecture emerge as key traits for the breeding of selected genotypes adapted to specific agro-ecological conditions. With our analysis, we aimed to provide the framework for initiating a *L. mutabilis* breeding program based on exploiting the available variation described herein. Finally, we reported potential biomass yields of up to 2 kg/plant of fresh biomass and 0.5 kg/plant of dry vegetative material in North European cropping conditions, suggesting that *L. mutabilis* could also find application as a source of lignocellulosic biomass, generating more than 50 t/ha of agricultural residues. The valuable use of the whole plant could significantly support and enhance the European bio-economy by supplying biomass from marginal lands, requiring no additional inputs. Future work should focus on assessing the quality of the biomass and testing its possible application in different bio-based products.

## Data Availability Statement

The original contributions presented in this study are included in the article/Supplementary Material, further inquiries can be directed to the corresponding author.

## Author Contributions

AG conceived and designed the experiments, analyzed the data, and wrote the manuscript. LT wrote the proposal, coordinated the project, and wrote and revised the manuscript. SA, DR, AM, B-JD, and JN-M coordinated the field trials and the collection of phenotypic data. AT, MG-R, and MT played an important role in ensuring access to South American germplasm material and data. M-JP provided support in the statistical analysis. All authors reviewed and approved the manuscript.

## Conflict of Interest

The authors declare that the research was conducted in the absence of any commercial or financial relationships that could be construed as a potential conflict of interest.

## Publisher’s Note

All claims expressed in this article are solely those of the authors and do not necessarily represent those of their affiliated organizations, or those of the publisher, the editors and the reviewers. Any product that may be evaluated in this article, or claim that may be made by its manufacturer, is not guaranteed or endorsed by the publisher.
