# Supplementary material for: Diversity and Agronomic Performance of Lupinus mutabilis Germplasm in European and Andean Environments
Source: Front Plant Sci. 2022 Jun 10;13:903661. doi: 10.3389/fpls.2022.903661 (PMC9226751; doi:10.3389/fpls.2022.903661)
Supplement: Supplementary file 1 [file Data_Sheet_1.docx]

Supplementary Material

# Supplementary Table

## Supplementary Table 1. Panel of 225 *L. mutabilis* accessions used in this study. Additionally, the cultivar I-450 Andino was used as reference in the Ecuadorian field trial.

The provider refers to the institution that provided the accessions: INIAP, Instituto Nacional de Investigaciones Agropecuarias from Ecuador; ISA, Instituto Superior de Agronomia of Lisbon, in Portugal; JKI, Julius Kühn-Institut in Germany; VDS, Vandinter Semo from The Netherlands.

| **LIBBIO code** | **Provider** | **Accession Code** | **Provenience** |
| --- | --- | --- | --- |
| LIB001 | INIAP | ECU-644 | Ecuador |
| LIB002 | INIAP | ECU-645 | Ecuador |
| LIB003 | INIAP | ECU-646 | Ecuador |
| LIB004 | INIAP | ECU-649 | Ecuador |
| LIB005 | INIAP | ECU-652 | Ecuador |
| LIB006 | INIAP | ECU-653 | Ecuador |
| LIB007 | INIAP | ECU-654 | Ecuador |
| LIB008 | INIAP | ECU-655 | Ecuador |
| LIB009 | INIAP | ECU-656 | Ecuador |
| LIB010 | INIAP | ECU-657 | Ecuador |
| LIB011 | INIAP | ECU-658 | Ecuador |
| LIB012 | INIAP | ECU-659 | Ecuador |
| LIB013 | INIAP | ECU-660 | Ecuador |
| LIB014 | INIAP | ECU-661 | Ecuador |
| LIB015 | INIAP | ECU-662 | Ecuador |
| LIB016 | INIAP | ECU-663 | Ecuador |
| LIB017 | INIAP | ECU-666 | Ecuador |
| LIB018 | INIAP | ECU-668 | Ecuador |
| LIB019 | INIAP | ECU-669 | Ecuador |
| LIB020 | INIAP | ECU-670 | Ecuador |
| LIB021 | INIAP | ECU-671 | Ecuador |
| LIB022 | INIAP | ECU-672 | Ecuador |
| LIB023 | INIAP | ECU-673 | Ecuador |
| LIB024 | INIAP | ECU-674 | Ecuador |
| LIB025 | INIAP | ECU-676 | Ecuador |
| LIB026 | INIAP | ECU-678 | Ecuador |
| LIB027 | INIAP | ECU-679 | Ecuador |
| LIB028 | INIAP | ECU-681 | Ecuador |
| LIB029 | INIAP | ECU-682 | Ecuador |
| LIB030 | INIAP | ECU-683 | Ecuador |
| LIB031 | INIAP | ECU-684 | Ecuador |
| LIB032 | INIAP | ECU-685 | Ecuador |
| LIB033 | INIAP | ECU-686 | Ecuador |
| LIB034 | INIAP | ECU-687 | Ecuador |
| LIB035 | INIAP | ECU-688 | Ecuador |
| LIB036 | INIAP | ECU-689 | Ecuador |
| LIB037 | INIAP | ECU-690 | Ecuador |
| LIB038 | INIAP | ECU-691 | Ecuador |
| LIB039 | INIAP | ECU-692 | Ecuador |
| LIB040 | INIAP | ECU-693 | Ecuador |
| LIB041 | INIAP | ECU-694 | Ecuador |
| LIB042 | INIAP | ECU-697 | Ecuador |
| LIB043 | INIAP | ECU-701 | Ecuador |
| LIB044 | INIAP | ECU-702 | Ecuador |
| LIB045 | INIAP | ECU-704 | Ecuador |
| LIB046 | INIAP | ECU-705 | Perú |
| LIB047 | INIAP | ECU-706 | Perú |
| LIB048 | INIAP | ECU-707 | Perú |
| LIB049 | INIAP | ECU-708 | Perú |
| LIB050 | INIAP | ECU-709 | Perú |
| LIB051 | INIAP | ECU-710 | Perú |
| LIB052 | INIAP | ECU-711 | Perú |
| LIB053 | INIAP | ECU-712 | Perú |
| LIB054 | INIAP | ECU-713 | Perú |
| LIB055 | INIAP | ECU-714 | Perú |
| LIB056 | INIAP | ECU-715 | Perú |
| LIB057 | INIAP | ECU-716 | Perú |
| LIB058 | INIAP | ECU-717 | Perú |
| LIB059 | INIAP | ECU-718 | Perú |
| LIB060 | INIAP | ECU-719 | Perú |
| LIB061 | INIAP | ECU-720 | Perú |
| LIB062 | INIAP | ECU-721 | Perú |
| LIB063 | INIAP | ECU-722 | Perú |
| LIB064 | INIAP | ECU-723 | Perú |
| LIB065 | INIAP | ECU-724 | Perú |
| LIB066 | INIAP | ECU-726 | Ecuador |
| LIB067 | INIAP | ECU-728 | Ecuador |
| LIB068 | INIAP | ECU-730 | Ecuador |
| LIB069 | INIAP | ECU-731 | Ecuador |
| LIB070 | INIAP | ECU-737 | Ecuador |
| LIB071 | INIAP | ECU-741 | Perú |
| LIB072 | INIAP | ECU-742 | Perú |
| LIB073 | INIAP | ECU-743 | Ecuador |
| LIB074 | INIAP | ECU-744 | Ecuador |
| LIB075 | INIAP | ECU-746 | Ecuador |
| LIB076 | INIAP | ECU-748 | Ecuador |
| LIB077 | INIAP | ECU-749 | Ecuador |
| LIB078 | INIAP | ECU-750 | Ecuador |
| LIB079 | INIAP | ECU-752 | Ecuador |
| LIB080 | INIAP | ECU-753 | Ecuador |
| LIB081 | INIAP | ECU-755 | Ecuador |
| LIB082 | INIAP | ECU-2332 | Ecuador |
| LIB083 | INIAP | ECU-2648 | Ecuador |
| LIB084 | INIAP | ECU-2651 | Ecuador |
| LIB085 | INIAP | ECU-2652 | Ecuador |
| LIB086 | INIAP | ECU-2653 | Ecuador |
| LIB087 | INIAP | ECU-2654 | Bolivia |
| LIB088 | INIAP | ECU-2655 | Ecuador |
| LIB089 | INIAP | ECU-2656 | Ecuador |
| LIB090 | INIAP | ECU-2657 | Perú |
| LIB091 | INIAP | ECU-2658 | Perú |
| LIB092 | INIAP | ECU-2660 | Perú |
| LIB093 | INIAP | ECU-2661 | Perú |
| LIB094 | INIAP | ECU-2664 | Perú |
| LIB095 | INIAP | ECU-2665 | Perú |
| LIB096 | INIAP | ECU-2667 | Perú |
| LIB097 | INIAP | ECU-2668 | Perú |
| LIB098 | INIAP | ECU-2669 | Perú |
| LIB099 | INIAP | ECU-2672 | Perú |
| LIB100 | INIAP | ECU-2673 | Perú |
| LIB101 | INIAP | ECU-2674 | Ecuador |
| LIB102 | INIAP | ECU-2675 | Ecuador |
| LIB103 | INIAP | ECU-2676 | Ecuador |
| LIB104 | INIAP | ECU-2678 | Ecuador |
| LIB105 | INIAP | ECU-2679 | Ecuador |
| LIB106 | INIAP | ECU-2680 | Ecuador |
| LIB107 | INIAP | ECU-2681 | Ecuador |
| LIB108 | INIAP | ECU-2682 | Ecuador |
| LIB109 | INIAP | ECU-2683 | Ecuador |
| LIB110 | INIAP | ECU-2684 | Ecuador |
| LIB111 | INIAP | ECU-2686 | Bolivia |
| LIB112 | INIAP | ECU-2688 | Bolivia |
| LIB113 | INIAP | ECU-2689 | Bolivia |
| LIB114 | INIAP | ECU-2691 | Bolivia |
| LIB115 | INIAP | ECU-2693 | Bolivia |
| LIB116 | INIAP | ECU-2697 | Bolivia |
| LIB117 | INIAP | ECU-2699 | Bolivia |
| LIB118 | INIAP | ECU-2700 | Bolivia |
| LIB119 | INIAP | ECU-2701 | Bolivia |
| LIB120 | INIAP | ECU-2702 | Bolivia |
| LIB121 | INIAP | ECU-2703 | Bolivia |
| LIB122 | INIAP | ECU-2704 | Bolivia |
| LIB123 | INIAP | ECU-2705 | Ecuador |
| LIB124 | INIAP | ECU-2706 | Ecuador |
| LIB125 | INIAP | ECU-2707 | Perú |
| LIB126 | INIAP | ECU-2708 | Perú |
| LIB127 | INIAP | ECU-2709 | Perú |
| LIB128 | INIAP | ECU-2710 | Perú |
| LIB129 | INIAP | ECU-2711 | Perú |
| LIB130 | INIAP | ECU-2712 | Perú |
| LIB131 | INIAP | ECU-2713 | Perú |
| LIB132 | INIAP | ECU-2714 | Perú |
| LIB133 | INIAP | ECU-2715 | Perú |
| LIB134 | INIAP | ECU-2716 | Perú |
| LIB135 | INIAP | ECU-2717 | Perú |
| LIB136 | INIAP | ECU-2718 | Perú |
| LIB137 | INIAP | ECU-2719 | Perú |
| LIB138 | INIAP | ECU-2720 | Perú |
| LIB139 | INIAP | ECU-2721 | Perú |
| LIB140 | INIAP | ECU-2722 | Perú |
| LIB141 | INIAP | ECU-2723 | Perú |
| LIB142 | INIAP | ECU-2724 | Perú |
| LIB143 | INIAP | ECU-2725 | Perú |
| LIB144 | INIAP | ECU-2726 | Perú |
| LIB145 | INIAP | ECU-2727 | Perú |
| LIB146 | INIAP | ECU-2728 | Perú |
| LIB147 | INIAP | ECU-2729 | Perú |
| LIB148 | INIAP | ECU-2730 | Perú |
| LIB149 | INIAP | ECU-2731 | Perú |
| LIB150 | INIAP | ECU-2733 | Perú |
| LIB151 | INIAP | ECU-2734 | Perú |
| LIB152 | INIAP | ECU-2735 | Ecuador |
| LIB153 | INIAP | ECU-2736 | Ecuador |
| LIB154 | INIAP | ECU-2738 | Perú |
| LIB155 | INIAP | ECU-2741 | Perú |
| LIB156 | INIAP | ECU-2743 | Ecuador |
| LIB157 | INIAP | ECU-2744 | Perú |
| LIB158 | INIAP | ECU-2746 | Ecuador |
| LIB159 | INIAP | ECU-2755 | Ecuador |
| LIB160 | INIAP | ECU-2756 | Bolivia |
| LIB161 | INIAP | ECU-2757 | Ecuador |
| LIB162 | INIAP | ECU-2758 | Perú |
| LIB163 | INIAP | ECU-2759 | Ecuador |
| LIB164 | INIAP | ECU-2760 | Ecuador |
| LIB165 | INIAP | ECU-2762 | Bolivia |
| LIB166 | INIAP | ECU-3048 | unknown |
| LIB167 | INIAP | ECU-3049 | unknown |
| LIB168 | INIAP | ECU-3050 | unknown |
| LIB169 | INIAP | ECU-3051 | unknown |
| LIB170 | INIAP | ECU-3052 | unknown |
| LIB171 | INIAP | ECU-3053 | unknown |
| LIB172 | INIAP | ECU-3054 | unknown |
| LIB173 | INIAP | ECU-3055 | unknown |
| LIB174 | INIAP | ECU-3056 | unknown |
| LIB175 | INIAP | ECU-3057 | unknown |
| LIB176 | INIAP | ECU-3058 | unknown |
| LIB177 | INIAP | ECU-3059 | unknown |
| LIB178 | INIAP | ECU-3060 | unknown |
| LIB179 | INIAP | ECU-3061 | unknown |
| LIB180 | INIAP | ECU-3062 | unknown |
| LIB181 | INIAP | ECU-3063 | unknown |
| LIB182 | INIAP | ECU-3064 | unknown |
| LIB183 | INIAP | ECU-3065 | unknown |
| LIB184 | INIAP | ECU-3795 | Ecuador |
| LIB185 | INIAP | ECU-5907 | Byelorussian Agricultural Academy |
| LIB186 | INIAP | ECU-5908 | Byelorussian Agricultural Academy |
| LIB187 | INIAP | ECU-5912 | Byelorussian Agricultural Academy |
| LIB188 | INIAP | ECU-5915 | Byelorussian Agricultural Academy |
| LIB189 | INIAP | ECU-5916 | Byelorussian Agricultural Academy |
| LIB190 | INIAP | ECU-5918 | Byelorussian Agricultural Academy |
| LIB191 | INIAP | ECU-5922 | Byelorussian Agricultural Academy |
| LIB192 | INIAP | ECU-7268 | Ecuador |
| LIB193 | INIAP | ECU-7269 | Ecuador |
| LIB194 | INIAP | ECU-7274 | Ecuador |
| LIB195 | INIAP | ECU-7276 | Ecuador |
| LIB196 | INIAP | ECU-7279 | Ecuador |
| LIB197 | INIAP | ECU-7280 | Ecuador |
| LIB198 | INIAP | ECU-7281 | Ecuador |
| LIB199 | INIAP | ECU-7282 | Ecuador |
| LIB200 | ISA | CM-157 | Portugal |
| LIB201 | ISA | I-82 | Portugal |
| LIB202* | ISA | INTI | Chile |
| LIB203 | ISA | LM-13 | Portugal |
| LIB204 | ISA | LM-18 | Portugal |
| LIB205 | ISA | LM231 | Portugal |
| LIB206 | ISA | LM-268 | Portugal |
| LIB207 | ISA | LM-27 | Portugal |
| LIB208 | ISA | LM-32 | Portugal |
| LIB209 | ISA | LM-34 | Portugal |
| LIB210 | ISA | LM-81 | Portugal |
| LIB211 | ISA | MUTAL | Portugal |
| LIB212 | ISA | P-20993 | Portugal |
| LIB213 | ISA | Potosi-Alem | Portugal |
| LIB214 | ISA | Potosi-ISA | Portugal |
| LIB215 | ISA | PRT79 | Portugal |
| LIB216 | ISA | SBP | Portugal |
| LIB217 | ISA | XM1-39 | Portugal |
| LIB218 | ISA | XM-5 | Portugal |
| LIB219 | JKI | JKL 210 | Germany |
| LIB220 | JKI | JKL 295 | Germany |
| LIB221 | JKI | JKL 309 | Germany |
| LIB222 | JKI | JKL 377 | Germany |
| LIB223 | VDS | Branco | Perú |
| LIB225 | INIAP | ECU-747 | Ecuador |
| LIB226 | INIAP | ECU-5913 | Byelorussian Agricultural Academy |
| I-450 ANDINO* | INIAP | I-450 ANDINO | Ecuador |

* Inti and I-450 ANDINO are lines that developed into cultivars.

## Supplementary Table 2. Decomposition of assessed variation for 16 morphophysiological traits in 225 *L. mutabilis* accessions over four different environments. Genetic, spatial and residual amount of variation are reported and used in the estimation of Broad sense heritability (H2). Seed yield assessment was divider over the different branching orders (MS= Main Stem, FO= first order, RO= Rest of orders). In the trial Portugal 2019, the count of Seeds and Pods RO included both seeds and pods from the first and rest of orders.

|  | native environment | | | | winter-MediterraneAN | | | | summer-North Central Europe | | | | | | | |
| --- | --- | --- | --- | --- | --- | --- | --- | --- | --- | --- | --- | --- | --- | --- | --- | --- |
|  | ECUADOR 2020 | | | | PORTUGAL 2019 | | | | NETHERLANDS 2019- clay | | | | NETHERLANDS 2020- sand | | | |
|  | Genetic | Spatial | Residual | H^2^ | Genetic | Spatial | Residual | H^2^ | Genetic | Spatial | Residual | H^2^ | Genetic | Spatial | Residual | H^2^ |
| Germination time | - | - | - | - | 0.98 | 2.21 | 2.63 | **0.51** |  |  |  |  | 22.86 | 0.02 | 16.29 | **0.78** |
| Height MS | 107.54 | 437.25 | 112.32 | **0.72** | 102.23 | 279.70 | 46.97 | **0.86** | 192.41 | 3.04 | 446.59 | **0.56** | 83.49 | 233.04 | 156.89 | **0.57** |
| Flowering time | - | - | - | - | 43.24 | 2.74 | 9.19 | **0.93** | 13.74 | 369.08 | 128.29 | 0.24 | 72.44 | 12.08 | 28.13 | **0.87** |
| Fresh biomass | - | - | - | - | 1299 | 10426 | 1644 | **0.68** | 7999 | 548128 | 95069 | 0.19 | 19282 | 695361 | 103853 | 0.32 |
| Branching  Orders | 0.02 | 0.39 | 0.14 | 0.33 | 0.03 | 0.52 | 0.10 | **0.44** | 0.05 | 0.87 | 0.18 | **0.46** | 0.01 | 0.10 | 0.47 | 0.07 |
| Pods MS | 1.46 | 18.78 | 4.28 | **0.48** | 3.79 | 64.63 | 14.68 | **0.42** | 5.52 | 0.86 | 3.24 | **0.83** | 2.75 | 0.12 | 8.23 | **0.46** |
| Pods FO | 5.17 | 125.28 | 63.24 | 0.18 | 20.96 | 1484.66 | 73.80 | **0.43** | 6.32 | 16.07 | 15.43 | **0.54** | 22.69 | 37.56 | 87.96 | 0.39 |
| Pods RO | 0.04 | 0.11 | 1.59 | 0.07 |  |  |  |  | 2.52 | 14.17 | 11.37 | 0.39 | 1.12 | 0.00 | 14.17 | 0.12 |
| Pods T | 10.92 | 212.41 | 100.11 | 0.23 | 26.52 | 707.11 | 102.75 | **0.41** | 29.28 | 32.09 | 37.33 | **0.69** | 40.66 | 42.20 | 80.87 | **0.58** |
| Seeds MS | 22.38 | 231.38 | 70.92 | **0.46** | 48.08 | 24.81 | 101.31 | **0.57** | 29.02 | 1.64 | 16.43 | **0.83** | 29.02 | 55.89 | 71.62 | **0.53** |
| Seeds FO | 29.05 | 823.24 | 459.55 | 0.15 |  |  |  |  | 25.00 | 75.66 | 57.50 | **0.55** | 142.48 | 150.12 | 240.18 | **0.62** |
| Seeds RO | 0.18 | 0.99 | 7.16 | 0.07 | 89.90 | 8279.82 | 472.17 | 0.33 | 7.93 | 23.18 | 38.13 | 0.38 | 0.00 | 1.71 | 7.63 |  |
| Seeds T | 78.77 | 1791.61 | 810.82 | 0.21 | 120.65 | 11258.51 | 583.84 | 0.36 | 115.09 | 168.36 | 135.03 | **0.7** | 296.77 | 381.62 | 445.17 | **0.64** |
| 100 Seed weight (g) | 4.32 | 0.20 | 5.82 | **0.67** | 6.76 | 841.85 | 90.53 | 0.17 | 4.71 | 11.63 | 8.08 | **0.63** | 64.52 | 39.95 | 527.97 | 0.24 |
| SEED YIELD  (g/plant) | 2.89 | 94.00 | 44.24 | 0.15 | 4.82 | 340.49 | 33.07 | 0.28 | 0.41 | 1.41 | 2.58 | 0.32 | 2.68 | 3.55 | 11.65 | 0.37 |
| Vegetative YIELD  (g/plant) |  |  |  |  | 107.7 | 984.7 | 237.3 | **0.54** | 975.42 | 187939.3 | 13114.81 | 0.17 | 958.33 | 2031.05 | 8493.03 | 0.21 |

# Supplementary Figure


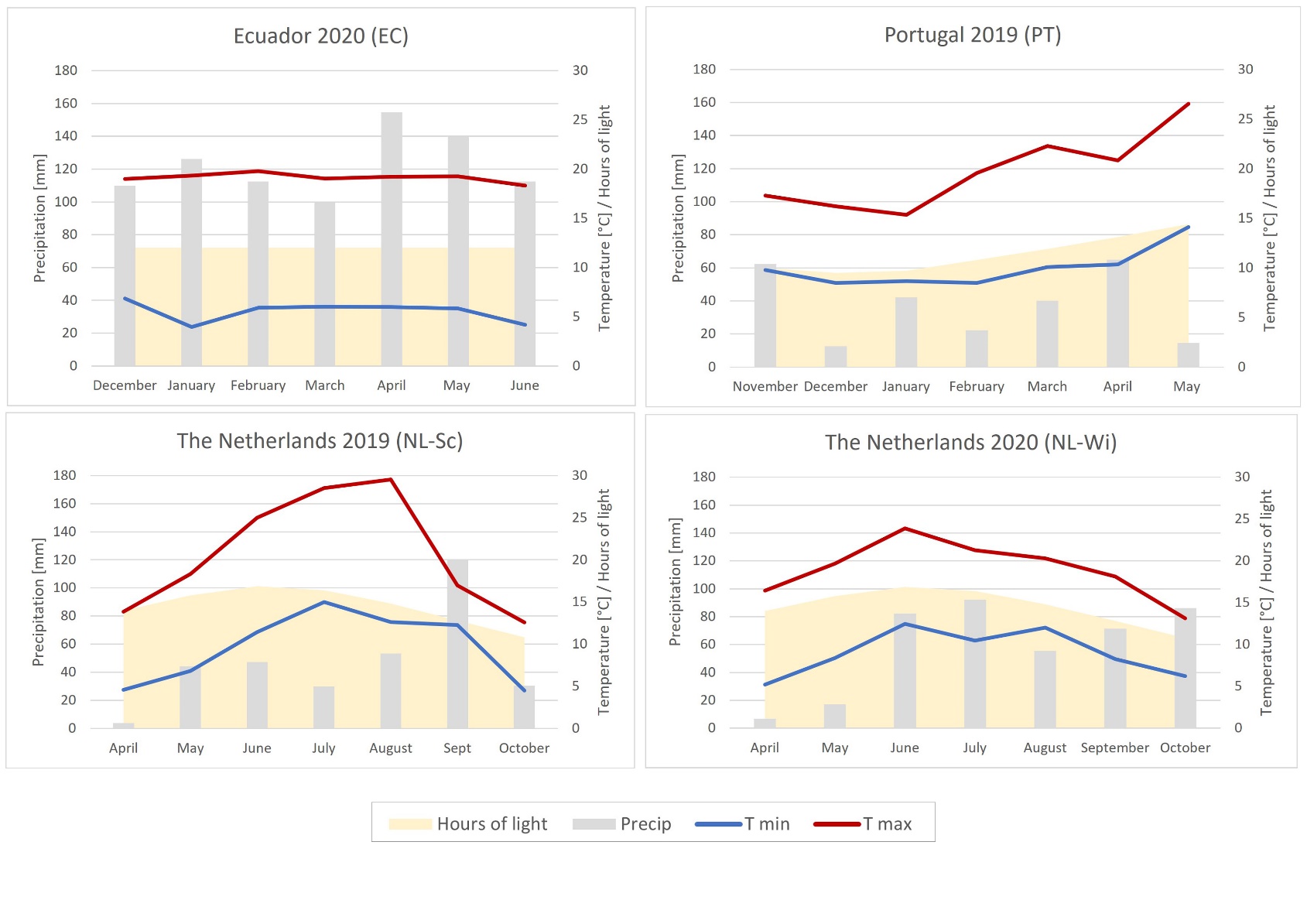
**Supplementary Figure 1.** Amount of precipitation (mm), Min and Max Temperatures (˚C) and Daylength (hours of light) in the four field trial locations across the cropping season.
